# Refined Detection and Classification of Knee Ligament Injury Based on ResNet Convolutional Neural Networks

**DOI:** 10.3390/life14040478

**Published:** 2024-04-05

**Authors:** Ștefan-Vlad Voinea, Ioana Andreea Gheonea, Rossy Vlăduț Teică, Lucian Mihai Florescu, Monica Roman, Dan Selișteanu

**Affiliations:** 1 Department of Automatic Control and Electronics, University of Craiova, 200585 Craiova, Romania; voinea.stefan.z5k@student.ucv.ro (Ș.-V.V.); monica.roman@edu.ucv.ro (M.R.); 2 Department of Radiology and Medical Imaging, University of Medicine and Pharmacy of Craiova, 200349 Craiova, Romania; ioana.gheonea@umfcv.ro (I.A.G.); lucian.florescu@umfcv.ro (L.M.F.); 3 Doctoral School, University of Medicine and Pharmacy of Craiova, 200349 Craiova, Romania; rossy.teica@gmail.com

**Keywords:** medical imaging, knee injury, anterior cruciate ligament, convolutional neural networks, ResNet, ACL-injury classification, 3D volume analysis

## Abstract

Currently, medical imaging has largely supplanted traditional methods in the realm of diagnosis and treatment planning. This shift is primarily attributable to the non-invasive nature, rapidity, and user-friendliness of medical-imaging techniques. The widespread adoption of medical imaging, however, has shifted the bottleneck to healthcare professionals who must analyze each case post-image acquisition. This process is characterized by its sluggishness and subjectivity, making it susceptible to errors. The anterior cruciate ligament (ACL), a frequently injured knee ligament, predominantly affects a youthful and sports-active demographic. ACL injuries often leave patients with substantial disabilities and alter knee mechanics. Since some of these cases necessitate surgery, it is crucial to accurately classify and detect ACL injury. This paper investigates the utilization of pre-trained convolutional neural networks featuring residual connections (ResNet) along with image-processing methods to identify ACL injury and differentiate between various tear levels. The ResNet employed in this study is not the standard ResNet but rather an adapted version capable of processing 3D volumes constructed from 2D image slices. Achieving a peak accuracy of 97.15% with a custom split, 96.32% through Monte-Carlo cross-validation, and 93.22% via five-fold cross-validation, our approach enhances the performance of three-class classifiers by over 7% in terms of raw accuracy. Moreover, we achieved an improvement of more than 1% across all types of evaluation. It is quite clear that the model’s output can effectively serve as an initial diagnostic baseline for radiologists with minimal effort and nearly instantaneous results. This advancement underscores the paper’s focus on harnessing deep learning for the nuanced detection and classification of ACL tears, demonstrating a significant leap toward automating and refining diagnostic accuracy in sports medicine and orthopedics.

## 1. Introduction

Anterior cruciate ligament (ACL) injuries are remarkably prevalent [1], particularly among individuals who engage in sports and activities demanding agility and quick changes in direction. These injuries are a common occurrence in the world of sports, with athletes in disciplines like soccer, basketball, football, and downhill skiing being particularly susceptible. The ACL, a critical ligament in the knee joint, serves as a key player in maintaining stability and preventing forward displacement of the tibia in relation to the femur [2]. This ligament is vulnerable to tears or strains when subjected to the high-impact and dynamic movements often required in these sports. The incidence of ACL injuries is not confined to professional athletes alone; they can affect individuals of all ages and activity levels [3]. With thousands of cases reported annually in many countries, ACL injuries continue to be a significant concern in the field of musculoskeletal health, necessitating continued research and efforts in prevention and treatment.

When an individual sustains a torn ACL, it can lead to a range of debilitating consequences [4]. A torn ACL can result in severe pain, swelling, and a significant loss of function in the affected knee. Mobility is often compromised, making it difficult to walk or engage in physical activities. Furthermore, a torn ACL can lead to long-term joint instability, increasing the risk of additional damage to the knee, such as meniscus tears or cartilage injuries. Untreated ACL injuries carry the potential for severe long-term consequences. If left untreated, they may lead to chronic pain, diminished joint function, and an elevated risk of developing osteoarthritis in the affected knee. Research indicates that the risk of osteoarthritis following an ACL injury is substantial, with estimates ranging from 20.3% to 37% at the 10-year mark. This risk escalates even further, reaching a range of 28.6% to 62%, after more than 20 years of follow-up [5]. These findings underscore the critical importance of accurate and timely diagnosis and treatment of ACL injuries to mitigate the potential for such adverse outcomes. Therefore, prompt diagnosis and appropriate medical intervention are crucial to address a torn ACL effectively and mitigate its long-term impact on an individual’s quality of life.

Arthroscopy and magnetic resonance imaging (MRI) are both valuable diagnostic tools for assessing ACL injuries, each with its own set of pros and cons. Arthroscopy, as an invasive procedure, provides a direct view of the knee joint and allows for the accurate assessment of ACL tears and related damage [6]. However, it involves surgical risks (infection, bleeding, blood clots, and nerve and blood vessel damage), can be uncomfortable (pain, post-procedure joint stiffness, and scarring), and requires anesthesia (allergic reactions and adverse effects). In contrast, MRI is non-invasive, making it a safer option, and it offers a comprehensive view of the knee’s internal structures [7]. It is useful for identifying other associated injuries. Hence arthroscopy is reserved nowadays mostly for people for whom MRI is not allowed or recommended due to various factors like the presence of metal implants, claustrophobia, pregnancy, severe obesity, inability to remain still, allergies or kidney function issues, age, and cooperation.

**Figure 1:**
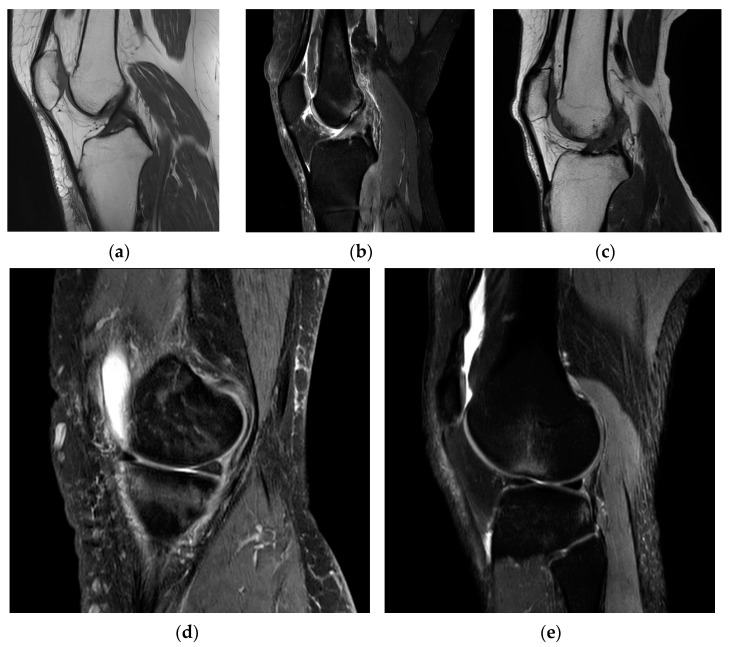
MRI spectrum of ACL conditions and associated features (part of the dataset): (**a**) normal ACL; (**b**) partially torn ACL; (**c**) torn ACL; (**d**) bone bruising in ACL tear; and (**e**) edema associated with ACL injury.

On an MRI scan in the sagittal plane, the appearance of the ACL can vary depending on its condition. In a healthy and intact ACL (Figure 1a), it typically appears as a well-defined, dark, and uniform band-like structure. It runs diagonally from the posterior aspect of the lateral femoral condyle (thigh bone) to the anterior aspect of the tibia (shin bone). The ligament should display a continuous and uninterrupted appearance on the MRI, indicating its normal structure [8].

In cases of a partial ACL tear (Figure 1b), the ligament may exhibit some changes in signal intensity. It might appear somewhat irregular or slightly brighter on the MRI, indicating mild damage to the ligament fibers.

When the ACL is completely torn (Figure 1c), it often appears discontinuous on the MRI. There may be a distinct gap in the ligament where it has torn, and this gap can be seen as an interruption in the dark, uniform appearance of the ligament. Swelling and surrounding tissue changes may also be evident. For example, bone bruising can be an indicator of an ACL injury (Figure 1d). This appears as bright or hyperintense areas on the MRI in the bone regions of the lateral femoral condyle and lateral tibial plateau due to the subluxation and impact associated with ACL tears. In instances of acute conditions, the presence of swelling or edema (Figure 1e), along with fluid accumulation, serves as an indicator of potential bleeding at that specific site.

The appearance of the ACL in the sagittal plane on an MRI T2 sequence is a critical component of diagnosing ACL injuries and assessing their severity by showing the clearest image of Blumensaat’s line [9]. Radiologists and orthopedic specialists carefully examine these images to determine the condition of the ligament and any associated damage, helping to guide treatment decisions.

This work represents an extension of the research presented in our previous paper [10]. In this extended work, improvements have been made in terms of both data quality and the methodologies employed for training and evaluation, with the overarching goal of attaining enhanced overall outcomes. More precisely, the current research introduces substantial advancements in dataset augmentation, training methodologies, and evaluation techniques aimed at enhancing the diagnostic precision of ACL-injury detection using MRI scans. Key enhancements include:

- Dataset expansion: The incorporation of an additional 602 MRI exams into our dataset, increasing the diversity and volume of data available for training and testing the model. This expansion allows for a more robust and comprehensive learning process, enhancing the model’s ability to generalize across a wider range of ACL-injury presentations;
- Refined two-stage training process: We have implemented a novel two-stage training methodology designed to first acquaint the model with a broad spectrum of ACL anatomy variations and subsequently fine-tune its focus on more subtle, detailed anatomical features. This approach ensures a more nuanced understanding of ACL injuries by the model;
- Tailored data augmentation: Different levels of data augmentation are applied across the two training stages. The initial stage employs a wide variety of augmentations to expose the model to a broad range of scenarios, while the second stage uses more targeted augmentations to concentrate on specific anatomical details and imaging artifacts. This strategic variation in augmentation helps in sculpting a more adaptable and accurate model;
- Stratified data split: ensuring each class is proportionally represented in both training and validation datasets through a stratified split addresses potential bias and improves the model’s reliability in real-world diagnostic scenarios;
- Selective model advancement: A selection mechanism filters the best-performing models from the first training stage to proceed to the second stage, based on their specific metrics. This ensures only the most promising models are refined further, optimizing the overall performance;
- Quality assurance of the dataset: By re-evaluating and correcting diagnoses for outliers identified in the original study, we have significantly improved the dataset’s quality. This meticulous quality-control process ensures the model is trained on accurate and clinically verified data, further enhancing its diagnostic accuracy.

Convolutional neural networks (CNNs) have emerged as the primary choice for image analysis, demonstrating their strength, particularly within the medical-imaging field. They frequently assist radiologists in the detection and classification of a range of health conditions [11,12,13,14,15,16,17,18,19,20,21]. A comparative review of these cited works [11,12,13,14,15,16,17] that tackle the diagnosis of ACL condition using deep neural networks is undertaken in Section 4, Discussions, where we examine their methodologies and findings in relation to our own study.

In this work, our goal is to provide a rapid and dependable diagnostic tool for ACL injuries that can serve as a preliminary benchmark. We channel the gathered data and associated labels into a two-phase training process, adapting a ResNet architecture to handle volumetric data. We track various metrics, including loss-function output, accuracy, F1 score, recall, precision, and ROC-AUC score. Employing a selection mechanism akin to that used in genetic algorithms, we advance only the most effective models from the first phase to subsequent training and refinement.

The contributions of this paper are summarized as follows:

- To our knowledge, it is the inaugural investigation to apply transfer learning to a 3D ResNet, originally pre-trained on non-medical videos, which is then further trained to differentiate among three severities of ACL injuries;
- It validates that our novel selection and training approach is advantageous for the fine-tuning of models that are top performers according to their specific metrics;
- The model undergoes comprehensive evaluation, utilizing not just a single training-evaluation approach but incorporating both Monte-Carlo and five-fold cross-validation techniques.

## 2. Materials and Methods

In our work, we present an enriched dataset from the University of Medicine and Pharmacy—Craiova’s Imaging Center, describing the acquisition of an extensive range of MRI scans and their classification into varying ACL conditions, with a focus on class and laterality distribution. Our methodological approach is detailed, highlighting the stratified division of data, innovative solutions for class imbalance, and advanced preprocessing techniques. We introduce an adapted 3D ResNet architecture, leveraging transfer learning from non-medical video data to enhance ACL-injury detection in MRI scans, underpinned by a comprehensive suite of performance metrics to validate the model’s efficacy.

### 2.1. Dataset

**Figure 2:**
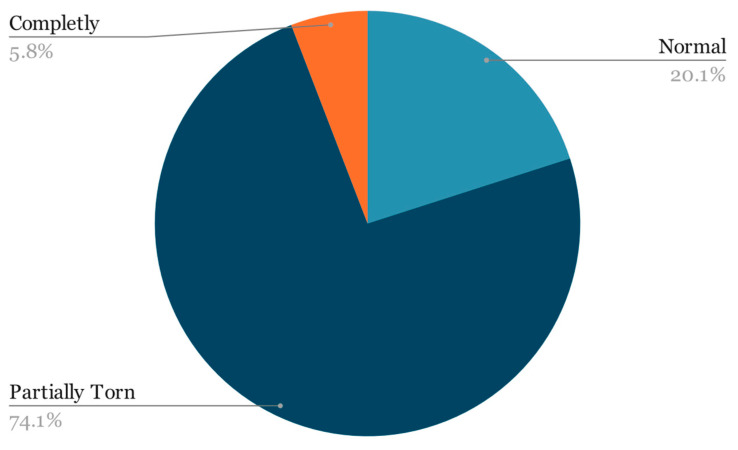
Class distribution.

For this research, the dataset mentioned in the prior paper was expanded by incorporating an additional year’s worth of examinations obtained by the University of Medicine and Pharmacy—Craiova’s Imaging Center, spanning from 2020 through the first half of 2023 via magnetic resonance analysis, summing up to a total of 1556 exams. The study was conducted retrospectively on patients who experienced knee pain under various circumstances, with the sole criterion being the proper execution of the MRI examination. The imaging data were acquired using a 3T Philips Ingenia MRI scanner, utilizing a magnetic field strength of 3.0 Tesla for each image acquisition. The data distribution among the 3 classes remains highly unbalanced (Figure 2):

- 91 completely torn ACLs;
- 1153 partially torn ACLs;
- 312 normal ACLs.

**Figure 3:**
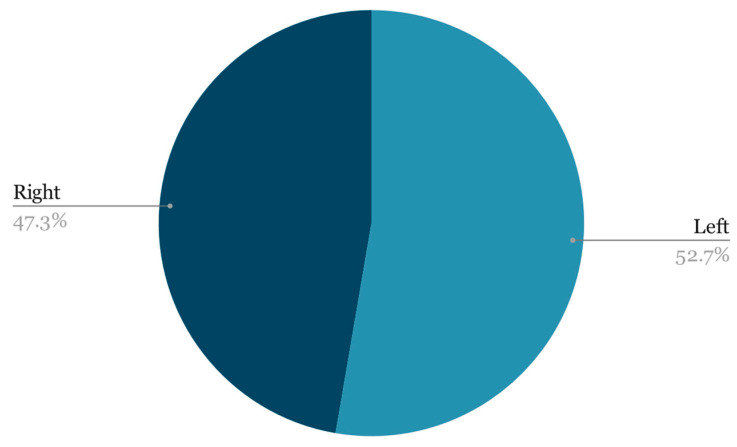
Laterality distribution.

When it comes to laterality, the two classes were quite evenly matched (Figure 3):

- Left 820;
- Right 736.

### 2.2. Method

**Figure 4:**
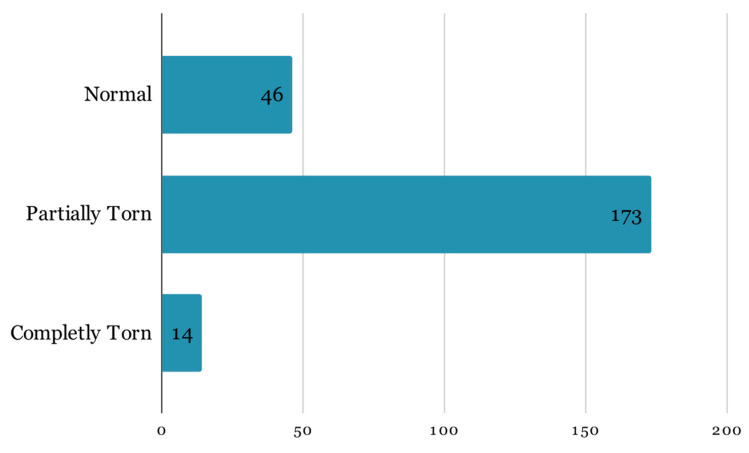
Dataset stratification—15%.

First, from the entire dataset, we split the training and validation dataset following a stratified split. Stratified sampling ensures that the distribution of classes in both the training and validation sets is representative of the overall dataset (Figure 4). Instead of randomly splitting the data, the dataset is divided in such a way that each subset contains a proportional representation of all classes [22].

Our decision to utilize an unconventional split of 85% training and 15% validation data (Figure 4) was driven by experiments and fine-tuning phases, particularly considering the challenges posed by our small and highly unbalanced dataset. This approach, while not common, was strategically chosen to maximize the amount of data available for training the model, which is crucial for learning robust features in scenarios where the dataset is limited. Furthermore, the highly unbalanced nature of our dataset necessitates a larger training set to ensure that the model is exposed to sufficient examples of the minority class, thereby improving its ability to generalize. This tailored split ratio has been empirically validated to enhance model performance and reliability in our specific context, indicating its suitability for small, unbalanced datasets.

Class imbalance is a pervasive issue in machine learning, particularly in medical datasets where certain conditions are naturally less common than others. In the context of ACL-injury diagnosis, the imbalanced dataset presents a significant challenge, as the prevalence of completely torn and normal ACLs is far lower than that of partially torn ACLs. This discrepancy can severely skew the training of a neural network, such as the ResNet model utilized in our study, leading to a bias towards the majority class.

**Figure 5:**
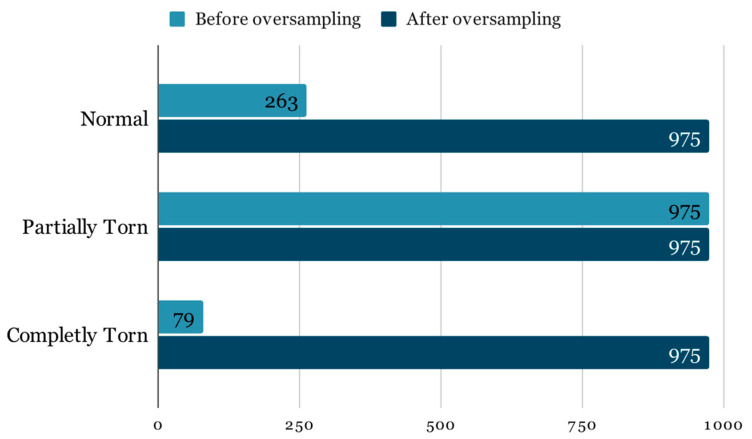
Class imbalance.

The Imbalance results in a model that may exhibit high overall accuracy, but this is misleading because the model may simply be predicting the majority class for all inputs. Such a model has limited clinical utility, as it fails to identify the less frequent, yet often more clinically significant, conditions. For instance, failing to correctly identify a completely torn ACL due to dataset imbalance could result in inadequate patient care. Therefore, it is crucial to employ in addition to the traditional accuracy evaluation metrics that can provide a more truthful representation of the model’s performance across all classes, such as the F1 score, precision-recall curves, or the area under the receiver operating characteristic curve (ROC-AUC). Fixing the imbalance by oversampling must be done with care to avoid introducing bias or overfitting to the minority class [23]. Overfitting occurs when the model learns patterns that are too specific to the training data, which do not generalize well to unseen data. It is crucial that the oversampling is applied exclusively to the training dataset. As illustrated in Figure 5, the adjusted class distribution post-oversampling depicts an equitable representation of each class, setting the stage for a more robust and generalizable training process.

In order to mitigate the possible drawbacks associated with oversampling the minority classes, which could include overfitting, information loss, evaluation bias, and reduced model generalization, we implemented image-preprocessing augmentation on the training data [24]. This involved applying the following transformations:

- Warp: This involves distorting the image in a non-linear manner, often simulating a perspective change or bending effects. This can help the model learn from images as if they were taken from different angles or perspectives;
- Sheer: Shearing shifts one part of an image to the side, tilting objects within the frame. It is akin to slanting an image horizontally or vertically, which can simulate a change in viewing angle relative to the object;
- Trapezoid: Similar to perspective distortion, this augmentation makes the image appear as if one end is farther away than the other, converting the rectangular shape of the image into a trapezoid. This can simulate a change in the object’s orientation relative to the viewer;
- Dihedral: Applies a combination of rotations and reflections to the image. There are eight possible transformations, including four rotations (0, 90, 180, 270 degrees) and flipping along one or both axes. This augmentation increases the variety of orientations the model is exposed to;
- Brightness: Adjusts the brightness level of the image, making it lighter or darker. This helps the model perform under various lighting conditions by training it to recognize features in both bright and dim scenarios;
- Contrast: Alters the contrast of the image, enhancing or reducing the difference between the lightest and darkest parts of the image. Adjusting contrast can help in highlighting or obscuring details, training the model to identify features across a range of contrast levels;
- Noise: Adds random variations of color or brightness to the images, simulating imperfections that can occur during image capture. This includes salt-and-pepper noise, Gaussian noise, etc. It helps the model to be more robust against grainy or slightly corrupted input images;
- Gaussian blur: Applies a blur effect to the image using a Gaussian function, creating a smooth, out-of-focus appearance. This simulates the effect of seeing the object at a distance or through an unfocused lens, teaching the model to recognize features without relying on sharp edges.

### 2.3. ResNet3D

Forming the understanding that MRI data inherently encodes volumetric information is straightforward, given that an MRI examination comprises a 3D volume that is then sliced into 2D images. Consequently, a conventional ResNet utilizing 2D convolutions is ill-suited for this task due to its incapacity to capture features extending along the Z-plane. The proposed architectural solution employs ResNet, short for residual neural network, a convolutional neural network architecture originally devised by Microsoft in 2015 for large-scale image classification tasks [25].

**Figure 6:**
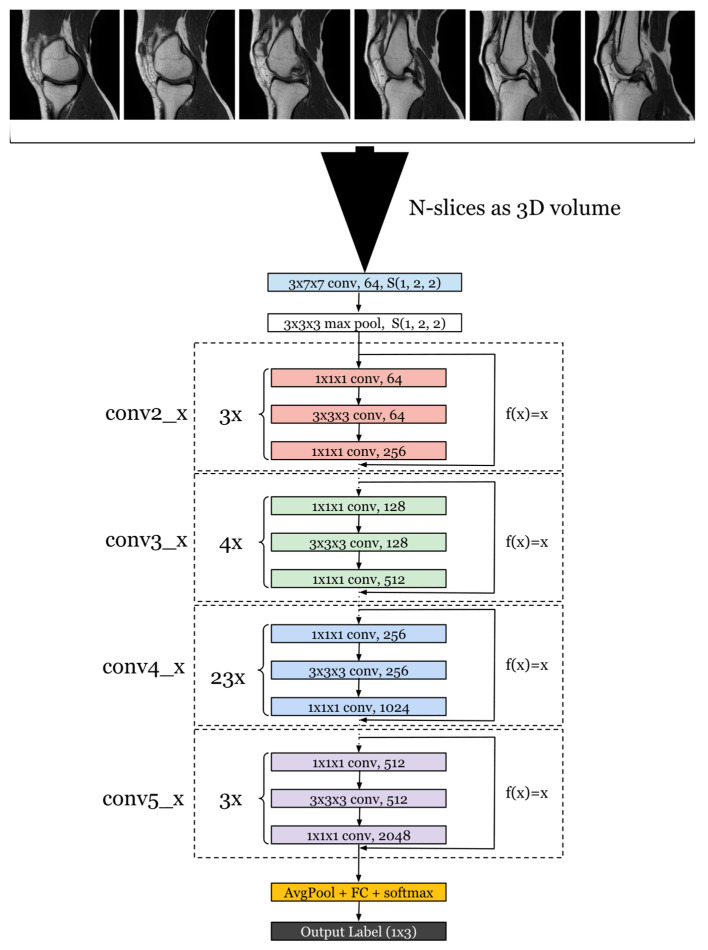
ResNet-101 adapted for 3D volumes.

To harness this 3D spatial context, we modified the ResNet architecture to utilize 3D convolutions [26] (Figure 6) instead of the standard 2D convolutions. This change allows the network to perform feature extraction not only on the plane of each slice but also across the slices, capturing the inter-slice spatial relationships that are imperative when dealing with volumetric data. This means that for each convolutional layer, the kernel moves along three axes, thereby analyzing a volume of the data at each step rather than a flat array.

Additionally, we increased the depth of the network to 101 layers, reflecting the deepest architecture available with pre-existing training, to allow for a more nuanced and complex representation of the data. This model possesses a substantial parameter count, totaling 87,314,176. By increasing the depth, the network can learn a hierarchical representation of the data with more abstract features being formed at higher layers. This is particularly beneficial when dealing with the complexity of MRI data, where differentiating between normal, partially torn, and completely torn ACL conditions requires the detection of subtle differences in the anatomy.

This transition to a 3D architecture necessitates adjustments in other layers as well. For example, the pooling layers are adapted to perform 3D pooling to maintain the 3D structure of the data throughout the network. The fully connected layers at the end of the network remain similar to the original ResNet-101 architecture, but they receive a flattened version of the 3D features extracted by the convolutional layers.

The shift to a 3D convolutional approach allows the model to better identify and classify the MRI images according to the degree of ACL injury. Each ACL condition presents unique features across the slices that may not be fully captured by a 2D analysis. By examining the full volume, our adapted ResNet3D-101 is able to provide a more accurate and reliable assessment of the ACL, leading to potentially better diagnosis and treatment planning for patients. The performance of this architecture is evaluated through a series of metrics that assess its ability to correctly classify the different ACL conditions, addressing both the accuracy of the classification and the model’s confidence in its predictions.

Despite the inclusion of more than a year’s worth of examinations in the dataset, training a model with randomly initialized weights is an impractical approach. Instead, we employed a model that had been pretrained on the University of Central Florida—101 (UCF-101) dataset, which comprises 101 action categories, each accompanied by a minimum of 101 video clips. These video clips depict a wide range of human actions, spanning sports activities, everyday motions, and various activities such as running, jumping, playing musical instruments, and more. It is noteworthy that the videos within the UCF-101 dataset vary in duration and quality, with some being relatively short and others being longer. Furthermore, since this dataset was sourced from YouTube, there exists variation in video quality and resolution.

In the process of analyzing MRI scans of the knee with the proposed architecture, our data loader is designed to package images that sequentially slice through the knee, typically at a specific distance apart, often in the range of 1 to 3 mm, depending on the resolution and specifics of the scan protocol. These slices are grouped into batches, which are then forwarded to the model for processing. Initially, the images are resized to a width (W) and height (H) of 112 × 112 pixels for the first stage of training, before being upscaled to 224 × 224 pixels for the second, finer training stage. Given that MRI images are inherently grayscale, our input only requires a single channel. However, to capture the full depth of the knee’s structure, we stack 20 such slices, creating a 3D volume represented in the input tensor dimensions as B × D × 1 × W × H. In the first stage, this equates to a tensor size of 60 × 20 × 1 × 112 × 112, reflecting a batch size (B) of 60. For the second stage, the tensor dimensions adjust to 8 × 20 × 1 × 224 × 224, corresponding to a reduced batch size of 8 to accommodate the increased spatial resolution. By employing this method, we leverage a pretrained model on video imagery, substituting the time dimension with depth to utilize 3D convolutions. This innovative approach allows us to emphasize features extending across multiple adjacent images, akin to how a classifier interprets a sequence of frames, thereby enhancing depth perception and feature detection within the complex anatomy of the knee. During inference, the batch size is set to 1 and directs our operations towards the finely tuned model. Consequently, the dimensions of the input tensor are 1 × 20 × 1 × 224 × 224.

This underscores the remarkable versatility and efficacy of transfer learning as a machine-learning technique. Transfer learning allows us to leverage the knowledge and representations learned from one domain or dataset and apply them to a different but related problem [27]. In the context of our research, the ability to take a model pretrained on the UCF-101 dataset and designed for action recognition in videos and apply it successfully to the task of ACL-injury detection is a testament to the power of transfer learning.

By doing so, we capitalize on the wealth of visual and spatiotemporal features learned from a diverse range of actions in the UCF-101 dataset. These features can be highly informative in recognizing patterns and anomalies within medical images, even though the source domain (action recognition) is dramatically different from the target domain (medical imaging). This efficient utilization of pre-learned knowledge not only saves time and computational resources but also often leads to more accurate and robust models for specific tasks.

It is a testament to the capacity of deep learning and neural networks to adapt and generalize across domains, making transfer learning a valuable tool for a wide array of applications, from computer vision to natural language processing and beyond. This ability to repurpose pre-existing models for new challenges greatly advances the state of the art in various fields, ultimately contributing to the development of more intelligent and capable AI systems.

To assess the model’s performance, we employed a range of metrics, including conventional accuracy, which quantifies the proportion of correct model predictions. Additionally, we considered the loss value on the validation set, as well as precision and recall. We also examined the receiver operating characteristic area under the curve (ROC-AUC) and the F1 score, which combines precision and recall to provide a comprehensive evaluation.

The ROC-AUC, or receiver operating characteristic area under the curve, provides valuable insight into the model’s ability to discriminate between positive and negative instances across various discrimination thresholds. This is particularly crucial in situations where you need to strike a balance between precision and recall or where the cost of false positives and false negatives varies depending on the application. The ROC-AUC’s ability to offer a comprehensive overview of the model’s performance under different operating conditions makes it a key tool in decision-making processes, helping to fine-tune model behavior to meet specific needs and constraints.

The choice of the multiclass cross-entropy loss function (1) is instrumental in training and evaluating the model. This loss function effectively quantifies the disparity between predicted class probabilities and the true class labels, making it particularly well-suited for multiclass classification tasks. It plays a pivotal role in guiding the model’s optimization process to minimize classification errors and improve its overall performance.

(1) $$-\overset{M}{\underset{c=1}{\sum }}y_{o,\;c}\log\left(p_{o,\;c}\right)$$

where *M* is the total number of classes, $y_{o,\;c}$ is a binary indicator if the class label c is the correct classification for the observation, and $p_{o,\;c}$ is the predicted probability that observation o belongs to class c. The log function in the formula heavily penalizes the model when it assigns a low probability to the true class. The more confident the model is in an incorrect prediction, the higher the loss.

## 3. Training and Results

Based on the specialized training regimen of the proposed neural network, we present a two-stage process designed to master the subtleties of MRI data for the accurate classification of ACL injuries. Initially, we harness a broad learning phase to capture a wide array of features, followed by an intensive fine-tuning phase to hone in on the finer details. The forthcoming sections will dissect these stages, revealing the intricate balance of hyperparameters, data augmentation, and architectural adjustments that culminate in a model primed for precision in medical diagnostics.

### 3.1. Two Stage Training

Following extensive GPU time spent on fine-tuning, the optimal training scheme was determined. It involves an initial phase of training for 30 epochs using the fit one cycle technique at a resolution of 112 × 112, followed by a subsequent refinement stage, where the model is trained at the full ResNet resolution of 224 × 224 for an additional 50 epochs.

The “Fit One Cycle” technique, often referred to as the “One-Cycle Learning Rate Policy”, is a training strategy used in deep learning for training neural networks, particularly convolutional neural networks (CNNs). It was introduced by Leslie N. Smith in his research paper titled “Cyclical Learning Rates for Training Neural Networks” [28]. The key idea behind this technique is to vary the learning rate during training in a cyclical or one-cycle manner to improve the model’s convergence and generalization.

Instead of using a fixed learning rate throughout the training process, the learning rate varies in a cyclical pattern. It starts at a relatively low value, gradually increases to a maximum value, and then gradually decreases back to the initial low value.

In addition to changing the learning rate, the technique often involves changing the momentum parameter during training. Momentum is a value that influences the step size of weight updates in the optimization process. It increased during the first half of the cycle and then decreased during the second half.

During the initial stage of training, we selected the largest batch size permissible within the memory constraints of our hardware, specifically the 24GB VRAM of the NVIDIA GeForce RTX 3090 GPU. This choice allowed for a batch size of 60 samples. The rationale behind this decision was two-fold: first, to expedite the coarse training phase, and, second, to simulate exposure to a diverse dataset through high-probability augmentations. This simulates a scenario where the model experiences a broad spectrum of data variance within each epoch, thus promoting a more robust feature extraction. For the fine-tuning phase, a batch size of eight was empirically determined to offer an optimal balance between training speed and convergence, ensuring precise model adjustments without sacrificing computational efficiency.

In the development of our methodology for classifying ACL-injury severity from MRI examinations, we considered the application of data-augmentation techniques as opposed to synthetic data generation. The rationale behind selecting data augmentation, and the specific percentages applied, stems from our aim to enhance the model’s ability to generalize across real-world variations without straying from the authentic distribution of clinical MRI data. Data augmentation introduces a controlled variability that mirrors actual differences encountered in clinical settings, such as variations in patient positioning, MRI machine calibrations, and soft-tissue contrasts. The chosen augmentation probabilities (Table 1) were calibrated in the parameters’ fine tuning to reflect realistic variations and ensure that the model is not merely memorizing the training data but learning to recognize the underlying anatomical features indicative of different ACL-tear levels.

Conversely, while data-generation techniques, such as generative adversarial networks (GANs), could potentially expand our dataset, they introduce the risk of generating synthetic anomalies that might not correspond to genuine clinical scenarios. This could inadvertently bias the model towards these non-authentic patterns, diminishing its clinical applicability and reliability. The percentages in Table 1 were thus determined through iterative experimentation, seeking a balance that enriches the dataset’s diversity without compromising the integrity of the medical images, as the model can become divergent and never generalize. This approach ensures that our model is trained on a dataset that not only challenges its learning capabilities but also closely aligns with the variability and complexity of real-world clinical MRI examinations.

In the initial training phase, greater emphasis is placed on applying augmentations with higher probabilities (Table 1) to the training images. This strategic approach ensures that the model is exposed to a more diverse range of features during the early training stages, thereby promoting enhanced generalization beyond the scope of the provided dataset. The second phase of training focuses on the fine-tuning process. During this stage, the augmentation probabilities are scaled down (Table 1), and the image size is enlarged.

These adjustments enable the model to detect finer details present in the MRI images. This phase employs both learning-rate slicing and weight decay. Learning-rate slicing is a dynamic method for identifying appropriate learning rates for fine-tuning pretrained models. It initiates with a base learning rate and applies a division factor at each epoch to establish a slice within the learning-rate range, as determined by the learning-rate finder. Weight decay, often referred to as L2 regularization, serves as a regularization method aimed at countering model overfitting. It incorporates an additional penalty component into the training loss function, with the purpose of promoting the restraint of model weights to lower magnitudes. This regularization technique is notably proficient in enhancing the model’s ability to generalize effectively.

The fine-tuning stage incorporated a weight decay factor of 1 × 10^−4^. This value was selected empirically, reflecting a common magnitude within the field, which serves to subtly penalize larger weight magnitudes without disproportionately impacting the learning trajectory. The magnitude of 1 × 10^−4^ for weight decay effectively strikes a balance between regularizing the model and maintaining the integrity of learned patterns, thus contributing to the prevention of overfitting while allowing for the retention of essential features in the model’s representations.

Out of the total of 50 epochs conducted during the fine-tuning phase, we employ a strategy to freeze the body of the model. This approach safeguards the retention of previously acquired features while directing our focus on training only the model’s head. The rationale behind this technique stems from the transition between the initial and second stages of training, where we elevated the resolution from 112 × 112 to 224 × 224. By first training the replacement head layer before refining the rest of the layers, we effectively manage the initial training process. The mechanism for saving checkpoints ensured that training did not continue beyond the point of optimal performance. We assured ourselves that training was not prematurely halted by monitoring the progression of the loss functions. This was evidenced by the stabilization of the validation loss and the onset of an increase in the training loss, signaling that the optimal generalization threshold had been reached. This approach ensures that the model successfully adapts to the new resolution while preserving the invaluable knowledge embedded in the pretrained layers.

### 3.2. Model Selection

To evaluate the model’s performance throughout training, we tracked several metrics:

- Validation loss, offering insights into the model’s generalization to unseen data;
- Training loss, quantifying the disparity between the model’s predictions and the actual target values on the training dataset;
- Accuracy, indicating the proportion of correctly classified instances among the total number of instances in a dataset;
- Precision, representing the ratio of correctly predicted positive instances to all instances predicted as positive. It gauges the model’s ability to minimize false positives, reflecting the precision or accuracy of positive predictions;
- Recall, measuring the proportion of actual positive instances correctly identified by the model. It provides an understanding of the model’s capability to capture all instances of a specific class, reducing false negatives;
- F1 score, accounting for both false positives and false negatives, providing a harmonic mean of precision and recall. Scaling from 0 to 1, a higher F1 score signifies a better balance between precision and recall, which is especially valuable when achieving equilibrium between minimizing false positives and false negatives is critical;
- ROC-AUC score, considering various classification thresholds and assessing the model’s ability to differentiate between classes. The ROC-AUC score is derived by plotting the true-positive rate (sensitivity) against the false-positive rate (one specificity) for different threshold values.

We integrated a callback listener to evaluate the model’s performance after each epoch based on the aforementioned metrics. If the model demonstrates improvement compared to the previously identified “best model”, we save a checkpoint. This approach is particularly valuable in conjunction with our two-stage training scheme. Following the initial training loop, we obtain the best models for each measured metric. The fine-tuning stage refines each of these models, resembling a genetic triage where selected models are further improved. In the second fine-tuning stage, at most six models are produced for each model from the first stage. We exclude the model with the best training loss so potentially overfitting models are not incentivized to be trained further. Epoch clashes, where more than one metric achieves its best, are trained only once to avoid redundant training of the same checkpoint or multiple mentions in the final results. Our initial assumption that finely tuned models could cater to specific cases was disproven by the findings presented in the results section of the second-stage data.

### 3.3. Results

**Figure 7:**
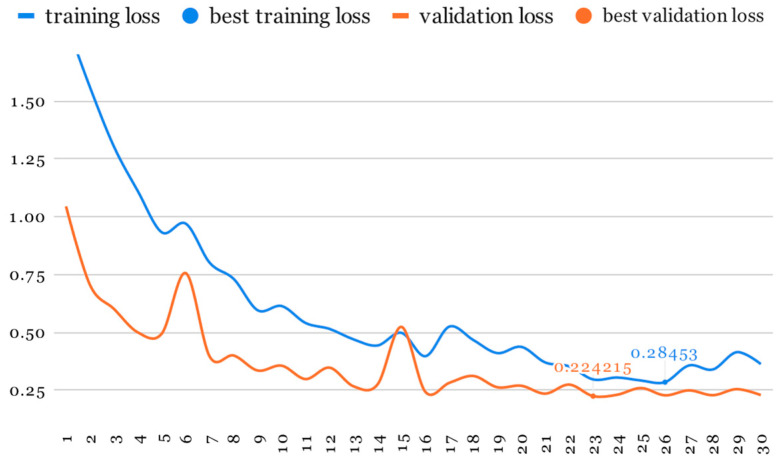
Loss evolution.

**Figure 8:**
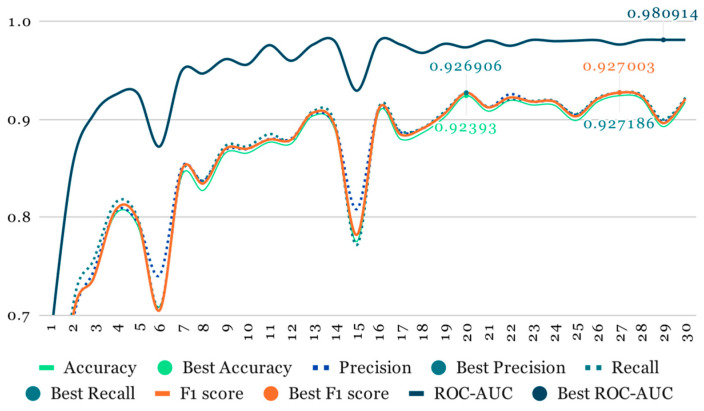
Metrics overview.

As mentioned earlier, our initial training phase involved using ResNet at half resolution (112 × 112, 20 slices) while applying a higher probability to all image-augmentation transformations (Table 1). This phase lasted for 30 epochs (Figure 7 and Figure 8).

Using the outcomes from the initial training phase, we identified the top-performing models for each recorded metric. Subsequently, these selected models underwent further fine-tuning training at the full ResNet resolution of 224 × 224 (20 slices) for an additional 50 epochs. Table 2 presents the best performance in each metric of the selected models.

**Figure 9:**
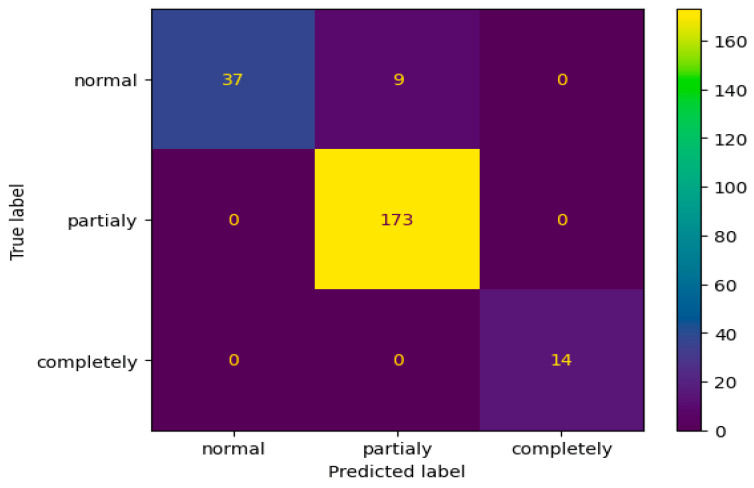
Confusion matrix.

Among the array of model variations stemming from the fine-tuning stage, the initial top performer in accuracy and recall emerged as the optimal choice across all monitored metrics. Consequently, we exclusively selected this model for printing the classification report (Table 3) and the confusion matrix from Figure 9.

In summary, the most successful model achieved is given in Table 4.

To rigorously assess the model’s performance on accuracy, F1, and ROC-AUC metrics, we conducted two comprehensive evaluations to uncover any potential weaknesses in our methodology.

The first of these evaluations employed the Monte-Carlo method (Table 5), which is a statistical technique that relies on repeated random sampling to obtain numerical results. Specifically, we maintained a validation–training split ratio of 15% to 85% and conducted the experiment across five iterations. Each iteration used a different seed to randomize the data split, effectively allowing us to evaluate the model’s performance across five unique 15%/85% splits. This approach helps in understanding the model’s stability and performance variability under different data distributions.

Following that, we implemented a five-fold cross-validation as a further step to validate the model (Table 6). In k-fold cross-validation, the dataset is evenly divided into k subsets, or ‘folds’. The model is then trained k times, each time using a different fold as the validation set, and the remaining k−1 folds are combined as the training set. This method ensures that every data point is used for both training and validation exactly once, providing a comprehensive overview of the model’s generalization ability across the entire dataset.

### 3.4. Limitations

The model, while rigorous in its approach and innovative in methodology, acknowledges certain limitations that are inherent to its design and scope:

- Data diversity: the dataset primarily comprises MRI scans from a specific demographic and machine type, which might limit the model’s generalizability to populations with different characteristics or scans obtained from varied MRI machines;
- Class imbalance solution: Although effective, the heavy reliance on data augmentation to address class imbalance raises questions about the model’s performance on purely original, non-augmented datasets. The predominance of augmented images, especially in underrepresented classes, might introduce biases or overfitting despite efforts to mitigate these effects;
- Model complexity: The two-stage training process, while beneficial for capturing detailed features, adds complexity to the model training and selection process. This complexity might not be easily replicable or may require significant computational resources, limiting its applicability in resource-constrained settings;
- Interpretability: The deep-learning nature of the model makes it a “black box”, where the specific features driving the classification decisions are not easily interpretable. This aspect might pose challenges in clinical settings where understanding the rationale behind a diagnosis is crucial;
- External validation: The current validation efforts, although extensive, are conducted on the same dataset split into different folds. External validation on completely independent datasets is necessary to fully assess the model’s generalizability and robustness across various clinical environments and patient populations.
- Long-term performance: The study does not address the model’s performance over time or its adaptability to evolving medical-imaging technologies and diagnostic criteria. Continuous learning or periodic retraining might be required to maintain its diagnostic accuracy;
- Specificity to ACLiInjuries: The focus on ACL injuries, while providing depth, also limits the breadth of applicability of the model. Expansion to other types of knee injuries or conditions could enhance the model’s utility in clinical settings.

## 4. Discussion

In prior research attempts, various authors have delved into the utilization of deep neural networks for ACL diagnosis via MRI-examination analysis. These studies explored diverse paths, investigating distinct architectural approaches, datasets, training methodologies, and related factors in pursuit of enhanced diagnostic outcomes.

The AlexNet architecture, historically renowned for its prowess in extracting image features, underwent pretraining with the Imagenet dataset [29] (Krizhevsky, Sutskever, and Hinton, 2012) and subsequent fine-tuning with MrNet images in the study titled “Deep-learning-assisted diagnosis for knee magnetic resonance imaging: Development and retrospective validation of MRNet” [11]. This approach resulted in area under the curve (AUC) values of 0.937, 0.965, and 0.847 for the respective assessments of detecting abnormalities, ACL tears, and meniscal tears. Despite the fact that the referenced study [11] focuses solely on two categories of ACL injuries, our comparison demonstrates significant improvements in ROC-AUC scores, with values of 0.991 in a custom data split, 0.989 in a Monte Carlo five-split evaluation, and 0.976 in a five-fold evaluation.

The research outlined in the paper [12] introduces an innovative architectural framework that integrates various neural networks, each specializing in distinct tasks. This method initiates with an initial phase termed “Slice Detection”, employing a LeNet convolutional neural network (CNN) to identify the slice where the ACL is prominently visible within the MRI images. Subsequently, the identified ACL slice is fed into the “Ligament Isolation” CNN based on the YOLO architecture [30], aiming to precisely extract the ACL region from the entire image. The isolated ACL image undergoes classification using the “Classification” DenseNet CNN, which assigns the relevant labels. This study announced an AUC of 0.98. This approach strategically bypasses the necessity of employing 3D convolutions, focusing solely on diagnosing within the ACL area. However, this method harbors limitations, notably the computational complexity associated with training three distinct models and architectures. Additionally, it is noteworthy that the ACL might not always be fully isolated within a single slice, often necessitating scrutiny across multiple slices for accurate diagnosis.

In the current study, we adopted a computationally efficient approach by utilizing a single model and architecture, deviating from the multi-model setup employed in the aforementioned research. This decision was made to streamline computational resources and enhance practicality.

While the previous study favored a slice-centric approach, our methodology seeks to leverage volumetric information comprehensively. We employ 3D convolutions to harness the full spectrum of three-dimensional data, allowing for a more in-depth analysis and potentially uncovering valuable insights within the MRI scans [20]. This strategic use of 3D convolutions aligns with our objective of achieving a comprehensive understanding of ACL injury in knee MR images. Compared to [12], by using a custom data split, we achieved a ROC-AUC score of 0.991, demonstrating our model’s effectiveness, which was further validated through Monte Carlo and five-fold cross-validation methods, obtaining similar values.

The paper titled “Efficient Detection of Knee Anterior Cruciate Ligament from Magnetic Resonance Imaging Using Deep Learning Approach” [13] addresses the non-binary classification problem of the ACL by employing a custom ResNet-14 convolutional neural network (CNN). To overcome the absence of 3D convolutions, the authors adopt a multi-stage configuration. Initially, the data undergoes preprocessing to extract the region of interest (ROI), which is subsequently rescaled to a standardized size of 75 × 75 using linear interpolation. Despite achieving accuracy rates of 92%, 91%, and 93% for normal, partial tear, and ruptured categories respectively, utilizing shallow custom models presents its own challenges. Notably, limitations include the inability to leverage transfer learning and the incapacity to capture intricate nuances within large datasets, restricting the exploration of certain edge cases.

In our approach, we emphasize the significance of transfer learning as a pivotal component in leveraging the capabilities of deep neural networks when working with limited datasets. Transfer learning enables us to harness the knowledge embedded in pre-trained models, even if those models were initially trained on data from a different domain. This practice provides our model with the advantage of starting with weights that have already undergone a prior training process, allowing it to discern valuable features efficiently and enhancing its convergence during subsequent training. This strategic utilization of pre-trained models aligns with our commitment to optimizing the capabilities of our approach and achieving robust results in the face of data limitations. The paper [13] also categorizes the three ACL-tear levels, similar to our approach. Our model, after undergoing a five-fold evaluation, exhibits a 1.2% increase in accuracy compared to the referenced study. This enhancement is substantial, considering it is at the high end of an already effective method, resulting in a 15% reduction in erroneous classifications.

“Effective automatic detection of anterior cruciate ligament injury using convolutional neural network with two attention mechanism modules” [14] paper presents a novel approach in the application of convolutional neural networks (CNNs) for detecting anterior cruciate ligament (ACL) injuries from MRI scans. By integrating two attention mechanism modules, somewhat similar to the ones used in advanced architectures suited for natural language processing (NLP), like transformers into their CNN architecture, the authors aim to enhance the model’s focus on relevant features within the MRI images, potentially increasing its diagnostic accuracy.

The methodology involves, as well, augmenting a dataset of MRI images from 313 patients, resulting in 630 pieces, including both injured and intact ACLs, to train and test the proposed model using five-fold cross-validation. The model achieves an average accuracy of 0.8064, with precision, sensitivity, specificity, and F1 scores of 0.7741, 0.9268, 0.6509, and 0.8436, respectively. The average area under the curve (AUC) for detecting an injured ACL is reported at 0.8886.

In our study, we also employed a sophisticated model architecture to tackle ACL-injury detection, achieving noteworthy results. Specifically, in our five-fold cross-validation, our model exhibited an average accuracy of 93.217%, with an average F1 score of 92.006% and a ROC-AUC of 0.97690. These results are comparatively higher than those reported in the study by Liang et al. [14], indicating a stronger performance in terms of both accuracy and reliability in detecting ACL injuries.

The difference in performance could be attributed to several factors, including the depth and complexity of the model architecture and the extent and variety of data augmentation techniques applied. Our model’s superior performance in five-fold cross-validation suggests that our methodological enhancements, possibly including the innovative approach to model training and the quality of the dataset, contribute significantly to the accuracy and robustness of ACL-injury detection.

In conclusion, while the study by Liang et al. [14] introduces an innovative approach by incorporating attention mechanisms into CNN for ACL-injury detection, the presented approach further advances the field by achieving higher diagnostic accuracy and reliability.

In the study of MRI analysis for knee injuries, Tsai et al. introduced the efficiently layered network (ELNet) [15], a custom CNN design tailored for diagnosing knee injuries via MRI. Their research highlights the significant impact automated tools have in streamlining the triage process for radiologists specializing in musculoskeletal disorders, aiding in the quicker identification of cases requiring further examination. A notable departure in their methodology is the decision to build the network from the ground up, without relying on transfer learning. This allows for a more specialized architecture that can be finely adjusted to meet the unique demands of knee MRI diagnostics, using either axial or coronal images as the input.

Our study takes a different approach by implementing a 3D ResNet architecture that has been pre-trained, contrasting with ELNet’s [15] choice to train from scratch. Additionally, our research benefits from a proprietary dataset, as opposed to ELNet’s [15] use of the MRNet database. This dataset encompasses a broader range of ACL-injury severities, including partial tears, enabling us to conduct a three-tier classification of knee injuries, a refinement over Tsai et al.’s [15] binary classification system. ELNet [15] reported achieving a ROC-AUC of 0.960 and an accuracy of 90.4%.

Another noteworthy study, “Automated detection of anterior cruciate ligament tears using a deep convolutional neural network” [16] applies the pretrained Xception [31] model, initially developed for ImageNet [29], fine-tuned with JPEG images derived from DICOM scans. This process introduces additional preprocessing steps, including image conversion to JPEG, which might degrade image quality, and image cropping to focus on relevant areas, increasing preprocessing time. This paper reported an accuracy of 86% and a ROC-AUC of 0.942.

In 2022, the paper [17] proposed a compact parallel deep convolutional neural network (CPDCNN), which features three parallel DCNN branches with various filter sizes, merging their outputs before the final classification layers. They explored different optimizers, with Adam yielding the best results: a 96.60% accuracy and an F1 score of 0.9610.

Our study’s results, achieved through a five-fold cross-validation, demonstrate the enhancements our methodology contributes to the domain, with a ROC-AUC of 0.9790, an accuracy of 93.217%, and an F1 score of 92.006%. These outcomes not only validate the benefits of integrating 3D convolutional layers and applying transfer learning but also emphasize the importance of a nuanced classification approach and the use of a comprehensive, specifically compiled dataset in advancing automated MRI-based diagnosis of knee-ligament injuries.

Following the publication of our work “Detection and Classification of Knee Ligament Pathology based on Convolutional Neural Networks” [10], we initiated efforts to delve into potential future work and enhancements. Among the identified limitations, we pinpointed an issue related to the quality of the labels. Given that these labels were extracted from diagnostic records authored by various medical professionals in a comprehensive manner, we acknowledged the potential for errors in this process and surmised that disparities might exist between the actual diagnosis and the extracted labels.

These disparities can be attributed to the parsing process, tasked with condensing intricate medical diagnosis narratives spanning multiple paragraphs into one of three potential labels. This categorization is solely accomplished by parsing the text for specific structures and keywords. In our opinion, this process presents a significant area where machine learning can play a pivotal role in substantial enhancements. By leveraging machine-learning techniques, the parsing procedure could undergo dramatic improvements, offering a more nuanced and accurate classification methodology for these complex medical diagnoses.

We examined misclassified instances by the CoDIT 2023 trained model [10], specifically focusing on cases with the highest losses. These instances with pronounced discrepancies in prediction were strong indicators that discrepancies might exist within the diagnosis labeling process. This scrutiny highlights the potential inaccuracies or ambiguities inherent in the current diagnostic labeling system. We began with those exhibiting a loss close to 1.0, indicating the model’s high confidence in its prediction despite the actual label being different. Our analysis extended to cases with a loss of 0.5, where the model expressed uncertainty by assigning a 50% probability to its prediction, leaving the remaining 50% distributed among the other two potential classes.

We shared a list of 73 cases with the radiology team at the University of Medicine, entrusting them with the task of meticulously re-evaluating and reassigning labels to ensure the datasets’ integrity. This relabeling process was intentionally conducted in a blind manner, safeguarding against any potential influence from the prior diagnoses. By removing any preconceived notions or biases, we sought to attain a more objective and reliable dataset for our ongoing research and analysis.

In Table 7 we present a concise overview of the reevaluation.

The results of the reevaluation strongly indicate the initial model’s effectiveness. The reassessment of 37 cases, carried out blindly, aligning with the model’s predictions, is a remarkable achievement. This outcome underscores the model’s accuracy and reliability in predicting the correct labels for these cases.

Building upon the foundation established in our previous work, this paper showcases significant advancements in our methodology and results. We have enriched our dataset both in scale and quality, leading to a marked improvement in model performance. Notably, accuracy has surged by 10% compared to our prior study, now reaching an impressive 97%. This leap in accuracy can be attributed to several key enhancements. First, the approach to partitioning the training and validation data has been refined with a stratified approach, ensuring a more representative and effective dataset split. This method has been instrumental in achieving a more balanced and comprehensive training process.

Additionally, the training methodology itself has undergone substantial refinement. From the initial stage, we retained the top-performing model for each observed metric. These models then progressed to the fine-tuning stage, where they were further optimized. This two-tiered approach to model selection and optimization has been pivotal in enhancing the overall performance.

Furthermore, we have employed advanced image transformations for data augmentation, significantly contributing to the robustness and generalizability of the model. This combination of dataset enhancement, methodical training, and sophisticated data-augmentation techniques underpins the notable leap in accuracy and the overall excellence of the model’s performance.

What is particularly striking is the scrutiny of the confusion matrix, revealing an intriguing observation: the model accurately distinguished between normal ACLs and torn ACLs without mislabeling any normal ones as torn or vice versa. The only misclassifications made by the model were in cases where nine examinations labeled as normal by radiologists were predicted to be partially torn. This occurrence is reasonable, considering the nuanced borderline between a partially torn ACL and a normal one, a distinction often challenging to delineate precisely.

## 5. Conclusions

The model exhibits commendable proficiency in effectively handling the classification task encompassing three distinct classes. While acknowledging the subjectivity inherent in these classes, the model showcases an exceptional ability to discern the intricate details that demarcate them. An intentional deviation from the conventional binary ACL labeling within radiology is embraced in this study, with a deliberate emphasis on the inclusion of a partially torn ACL class. This distinctive classification holds the potential to significantly contribute to identifying cases amenable to non-surgical intervention. The primary objective of this paper is not to supplant the role of radiologists but to streamline the initial diagnostic process for patients in a highly efficient and cost-effective manner.

Furthermore, the proposed model demonstrates promise in expediting the establishment of a first baseline diagnosis. This computational framework holds potential for deployment in initiating clinical trials, thereby presenting an avenue for pragmatic implementation within clinical settings.

For future research, we plan to initiate a clinical trial to rigorously test the ACL-tear-level classification model. The trial will be conducted by integrating the model into the diagnostic process at selected medical facilities. Radiologists and orthopedic specialists will use the model’s outputs as part of their diagnostic toolkit, comparing its efficacy to conventional diagnosis methods. Key performance indicators such as accuracy, sensitivity, and specificity will be tracked to evaluate the model’s performance in a clinical setting. Additionally, we aim to establish a feedback loop where the model is continuously updated. On a weekly basis, it will be fine-tuned with newly acquired MRI exams to adapt to new data and potentially improve its predictive capabilities. This approach ensures the model remains relevant and accurate over time, adapting to any variations in imaging techniques or patient demographics. Through this iterative process, we expect to not only validate the model’s current efficacy but also enhance its utility in clinical diagnostics.

## Figures and Tables

**Table 1:** Data augmentation.

Image Augmentation	1st-Stage Probability	2nd-Stage Probability	Parameters
Warp	10%	2%	magnitude [−0.25, 0.25]
Sheer	10%	2%	magnitude [−0.25, 0.25]
Trapezoid	10%	2%	magnitude [−0.25, 0.25]
Dihedral	10%	2%	random number [0, 17] determines flipping and transposing dimensions
Brightness	20%	5%	beta_range [−0.3, 0.3] with 25 steps
Contrast	20%	5%	alpha_range [0.7, 2.0] with 25 steps
Noise	20%	5%	standard_range [0.01, 0.1]
Gaussian blur	20%	5%	random kernel size between {5, 11} with sigma 0.5

**Table 2:** Progressive model metrics summary.

First Stage			Fine-Tuning Stage	
Epoch	Metric	Value	Metric	Recorded Value @ Epoch
19	Accuracy	0.92393	ROC-AUC	**0.99101 @ 28**
			Validation loss	**0.13213 @ 41**
	Recall	0.926906	Accuracy	**0.97147 @ 49**
			Precision	**0.97422 @ 49**
			Recall	**0.97187 @ 49**
			F1 score	**0.97238 @ 49**
22	Validation loss	0.22422	ROC-AUC	0.98187 @ 20
			Validation loss	0.19888 @ 31
			Accuracy	0.96038 @ 47
			Precision	0.96319 @ 47
			Recall	0.96191 @ 47
			F1 score	0.96140 @ 47
26	F1 score	0.92700	ROC-AUC	0.98527 @ 34
			Validation loss	0.17472 @ 36
	Precision	0.927186	Accuracy	0.96197 @ 46
			Precision	0.96470 @ 46
			Recall	0.96236 @ 46
			F1 score	0.96327 @ 46
28	ROC-AUC	0.98091	ROC-AUC	0.98750 @ 24
			Validation loss	0.19032 @ 30
			Accuracy	0.95087 @ 40
			Precision	0.95493 @ 40
			Recall	0.95272 @ 40
			F1 score	0.95353 @ 40

**Table 3:** Classification model performance metrics.

	Precision	Recall	F1 Score	Support
0	1.00	0.80	0.89	46
1	0.95	1.00	0.97	173
2	1.00	1.00	1.00	14
accuracy			0.96	233
macro avg	0.98	0.93	0.96	233
weighted avg	0.96	0.96	0.96	233

**Table 4:** Overall performance.

Metrics	Value
Accuracy	97.147%
F1 score	97.238%
ROC-AUC	0.99101

**Table 5:** Monte Carlo evaluation.

	Average	1st Split	2nd Split	3rd Split	4th Split	5th Split
Accuracy	96.323%	97.147%	95.404%	96.989%	96.197%	95.880%
F1 Score	96.416%	97.238%	95.504%	96.941%	96.346%	96.051%
ROC-AUC	0.98911	0.99101	0.98417	0.99043	0.99194	0.98801

**Table 6:** Five-fold evaluation.

	Average	1st Fold	2nd Fold	3rd Fold	4th Fold	5th Fold
Accuracy	93.217%	94.550%	92.411%	92.741%	93.698%	92.682%
F1 Score	92.006%	92.196%	92.849%	93.148%	89.094%	92.746%
ROC-AUC	0.97690	0.99036	0.97267	0.96896	0.98335	0.96919

**Table 7:** Diagnostic reassessment and dataset curation for MRI analysis.

Reevaluation	Number of Cases	Action
Bad examination—the original diagnosis was set by checking other MRI sequences	4	Remove from the dataset any MRIs that are of poor quality or depict ligaments that have undergone surgical intervention, as the model cannot effectively generalize from such cases.
Ligamentoplasty already done	3	
Advanced degenerative changes	4	Retained within the dataset to allow the model to acquire knowledge of these features as part of its learning process.
Large fluid buildup	5	
Reclassified and matched the predicted label	37	Kept in the dataset
Reevaluation kept the label different from the predicted one	20	

## Data Availability

The data presented in this study are available on request from the corresponding author. The data are not publicly available due to privacy.
